# Report of the Committee of Internal Health on the Asiatic Cholera, Together with a Report of the City Physician of the Cholera Hospital

**Published:** 1850-07

**Authors:** 


*Report of the Committee of Internal Health on the Asiatic Cholera, together with a Report of the City Physician of the Cholera Hospital.* Boston, 1849. 8vo. pp. 183.

The Report of the Committee of Internal Health, in Boston, is a well written sketch of the condition of the city at the time it commenced its labors, and of the measures adopted by it to prepare for the visitation of the cholera, by a thorough investigation of all the causes prejudicial to the public health, with the means adopted for their removal. In this arduous work they were ably sustained by the physicians, and it is through their joint labors that we are presented with a most complete and perspicuous account of the localities in which the cholera chiefly prevailed. A number of excellent wood-cuts of the principal places in which it exercised its ravages, and a ground plan of the city, accompany the

Report. We confess that, before reading this Report, we were ignorant that, in so cleanly a city as is Boston, such abodes of wretchedness as those described, could be found. We take the following as an example ; it is a description of Half Moon Place, " probably the worst locality in the city."

" The houses are built around an area from which air is almost totally excluded by the perpendicular wall of Fort Hill on one side, and the lofty buildings of Broad street on the other. A large part of the area is occupied by some twelve or fourteen privies, constantly overflowing, and by ill-constructed and worn out sinks and drains, into which are hourly thrown solid substances, of all sorts, which choke them up, and cause the liquid parts mixed with them to run over. Into the area there is a narrow entrance from Broad street, whilst a steep and crazy stair-case affords a passage to Humphrey place, some fifty feet above. Side by side with the stair-case, and fully exposed, a large, square plank drain makes a precipitous descent, conducting, half hidden, half revealed, not only the waste water of the houses in Humphrey place, but also the contents of its privies to the area below, which, as may be supposed, is redolent of the fact."

It can hardly be a matter of surprise, that the wretched tenants of these filthy localities, were rapidly swept away by the merciless epidemic. The City Hospital was established in this vicinity, but it is stated that it did not itself become a focus of contagion, and its immediate neighborhood having been completely purified, escaped the epidemic entirely.

The medical report is drawn up by Dr. Clark, from the materials furnished by his colleagues and assistants ; the manner in which it is executed, and the great number of interesting facts it contains, reveal the most persevering industry on the part of these gentlemen, especially that portion descriptive of the morbid appearances found after death. Too much praise cannot be awarded them, for the great accuracy with which their autopsies were conducted, in view of the all-engrossing duties of the staff of a cholera hospital, and the negative results which it was anticipated the examinations would yield. It must be owned that they afford us little insight into the true nature of the disease, and still less do they offer any indications for an appropriate treatment ; nevertheless we would not undervalue the importance of any facts, fully attested, however obscure their relations may at present be to us.

While, therefore, as is stated in the Report, the most remarkable and constant appearances were, an unusual dryness and stickiness of the serous membranes, a moderate tumefaction of Peyer's glands, and the "glandulæ solitariæ," and a shrivelled condition of the spleen, and these may be probably regarded as characteristic phenomena found only in cholera patients—we are not without hope, that the mutual dependancy of symptoms and lesions may at some day become more manifest than they are at present. It is hardly possible that thirty-two carefully conducted autopsies of cholera patients, shall not be of some value in the elucidation of the disease, and have some indirect bearing upon its curative treatment.

The results obtained by Dr. Clark and his colleagues agree pretty closely with those arrived at by Dr. Gairdner of Edinburgh, and which were quoted in the American Journal for October, 1849. The absence of those appearances of congestion, so commonly loosely assumed to exist in cholera, as well as of any viscidity of the blood, the occurrence of ecchymoses in various parts, and particularly in the sub-mucous coat of the intestines, and the presence of bile in the gall-bladder and sometimes in the intestines, as in persons dying of other diseases, are all points which receive ample confirmation from the reports of both observers. Dr. Clark differs, however, from Dr. Gairdner, in his opinion that the abundance of uninjured epithelium cells found floating in the intestinal fluids after death from cholera, is due to a separation of the same by the maceration of the mucous coat of the bowel. The only fact of importance, alleged by him in support of this difference of opinion, is, that the mucous surface of the vagina and of the bladder, neither of which are, of course, subjected to maceration, are covered uniformly with a "thick white pasty or creamy secretion, which, on microscopic examination, is seen to consist entirely of detached epithelium cells, mostly perfect in shape and generally distinctly nucleated." While the question remains still *sub judice*, the important fact announced by Dr. Gairdner, "that the true cholera stools *passed during life*, contained so little perfect epithelium, that it cannot be considered as anything more than an accidental ingredient," renders the idea very improbable, that the exfoliation spoken of can play a very important part in the disease. The reality of this exfoliation, (whether it be before or after dissolution,) is most clearly demonstrated by the injections of Dr. Neill, presented to the museum of the College of Physicians of Philadelphia, with the Report of the Committee appointed by that body, "to examine into the condition of the mucous membrane of the intestinal canal in persons dying of cholera." In the language of that Report, "The epithelial layer of the intestinal mucous membrane, was in all specimens either entirely removed, or was detached, adhering loosely as a pulpy layer, mixed with mucous or an albuminoid substance." The tumefaction of the solitary and Peyer's glands was observed also by this committee as well as by Dr. Clark. A very interesting case is related in Dr. Clark's Report, (p. 50,) of a patient who passed into the typhoid condition so often following cholera, and died after it had lasted a week, or from the invasion of the cholera, ten days. "The patches of Peyer, situated at the lower part of the ileum, were inflamed and deeply ulcerated; two of them showing an abundant deposit of typhus matter, which projected into the intestinal cavity, like loose, granular coagula." The spleen, too, was engorged and enlarged, and the remaining anatomical conditions were not dissimilar from those found in spontaneous typhoid fever. If this patient was really in health before he was attacked with cholera, the case is certainly a most uncommon one, and deserved to be given in more detail than it is presented to us.

The treatment pursued in the Hospital was very unsuccessful, and that for the very evident reason which may be predicated of all public hospitals in times of pestilence, viz. that the patients were not brought in, until it was beyond the power of medicine to be of any service to them. During a period of five months and a half, two hundred and sixty-two patients were "*subjected to treatment*," of these one hundred and sixty-six died, and ninety-six recovered. "Patients came in, so far gone in collapse, that we expected them to die at any rate. With this feeling it was in several instances judged best to give them no medical treatment, but to let them follow their own inclinations. In no one case where this practice was followed, did the patient recover."

"Apparent narcotism," the Report says in another place, "was noticed in many of the fatal cases upon entrance. The patients who exhibited this symptom had generally been many hours under treatment before entrance, and from many of them we learned that excessive doses of opium had been taken. In most of these patients there was a contracted pupil, not influenced by light or darkness, stertorous breathing, difficulty of hearing, together with the customary evacuations and occasional cramps. These appearances were the more remarkable, because in the generality of fatal cases, patients were decidedly active and intelligent until within a short time of death, which in these cases was always the speedy result.

The Report gives a very discouraging view of the utility of remedies. It condemns, either as useless or injurious, narcotics, camphor, stimulants, calomel, emetics, quinia, astringents, aromatics and cathartics, ether, venous injections, external heat, dry or moist, and " packing" in the wet sheet. The only remedies favorably spoken of, as palliatives, or useful at certain stages of the disease, are the cold sponge baths, cold affusion, bleeding, wood naphtha, and saline medicines. There is unfortunately no comparative tabular or other statement given of the remedies used in the cases which recovered, and those which died. The medical staff of the Hospital must have been reduced to the verge of despair by the inefficiency of their treatment, when they seriously thought of trying the homœopathic practice. But fortunately, we think, " no one of our number understood it, and notwithstanding offers were made to several homœopathic practitioners, we could not find among those any who were willing to come into the Hospital, upon equal terms, and take charge of an equal number of patients with ourselves." If the homœopathic practitioners have faith in their doctrine and are not afraid to reveal the mysteries of their practice to the eyes of physicians, let them build their own hospitals and practice their system on as many as may voluntarily submit to it; but for our part, we must protest against the spirit of weak liberality shown in the above instance. We cannot but feel that it is derogatory to that love of truth which should characterize the medical profession, to entrust human life in the hands of those who practice an ephemeral folly, equally irreconcileable with truth or common sense.

The numerous facts contained in these Reports, render them extremely valuable, and we therefore heartily commend them, especially to those interested in the study of the history of the late epidemic.

